# Comprehensive analysis of mitophagy-related genes in NSCLC diagnosis and immune scenery: based on bulk and single-cell RNA sequencing data

**DOI:** 10.3389/fimmu.2023.1276074

**Published:** 2023-12-14

**Authors:** Haibo Yu, Qingtao Liu, Mingming Jin, Gang Huang, Qianqian Cai

**Affiliations:** ^1^ School of Health Science and Engineering, University of Shanghai for Science and Technology, Shanghai, China; ^2^ Shanghai Key Laboratory of Molecular Imaging, Shanghai University of Medicine and Health Sciences, Shanghai, China; ^3^ School of Medical Imaging, Shanghai University of Medicine and Health Sciences, Shanghai, China; ^4^ Department of Cardiothoracic Surgery, Xinhua Hospital Affiliated Shanghai Jiao Tong University, School of Medicine, Shanghai, China

**Keywords:** non-small cell lung cancer, mitophagy-related genes, prediction model, subtyping, immune infiltration, immunotherapy response

## Abstract

Lung cancer is the main cause of cancer-related deaths, and non-small cell lung cancer (NSCLC) is the most common type. Understanding the potential mechanisms, prognosis, and treatment aspects of NSCLC is essential. This study systematically analyzed the correlation between mitophagy and NSCLC. Six mitophagy-related feature genes (*SRC*, *UBB*, *PINK1*, *FUNDC1*, *MAP1LC3B*, and *CSNK2A1*) were selected through machine learning and used to construct a diagnostic model for NSCLC. These feature genes are closely associated with the occurrence and development of NSCLC. Additionally, NSCLC was divided into two subtypes using unsupervised consensus clustering, and their differences in clinical characteristics, immune infiltration, and immunotherapy were systematically analyzed. Furthermore, the interaction between mitophagy-related genes (MRGs) and immune cells was analyzed using single-cell sequencing data. The findings of this study will contribute to the development of potential diagnostic biomarkers for NSCLC and the advancement of personalized treatment strategies.

## 1 Introduction

According to the GLOBOCAN 2020 report compiled by the International Agency for Research on Cancer (IARC), lung cancer is ranked the second most prevalent cancer worldwide and remains a leading cause of cancer-related mortality (1). It can be classified into two main types: non-small cell lung cancer (NSCLC), which accounts for approximately 85% of cases, and small cell lung cancer (SCLC), which accounts for approximately 15% of cases. The prognosis of lung cancer is often unfavorable due to factors such as late-stage diagnosis and resistance to radiation therapy and chemotherapy. However, recent advancements in targeted therapy and immunotherapy have shown promising results in reducing mortality rates (2, 3). Consequently, there is a growing need for the development of reliable and accurate prognostic biomarkers using high-throughput technologies to assist clinicians in optimizing treatment strategies.

Mitochondria are essential organelles that serve as the primary source of cellular energy and play a vital role in various cellular processes, including adenosine triphosphate (ATP) synthesis through oxidative phosphorylation (OXPHOS). Impaired mitochondrial function reduces the OXPHOS capacity, which results in the elevated production of reactive oxygen species (ROS) and subsequent cellular damage. Uncontrolled mitochondrial oxidative stress has been implicated in the development of various diseases, including cancer (4, 5). Additionally, mitochondria are closely associated with metabolism, cell signaling, and programmed cell death. Various triggers, including oxidative stress, DNA damage, and imbalances in calcium regulation, lead to apoptosis via this mitochondrial route (6). Moreover, excessive ROS production by mitochondria can promote lipid peroxidation, leading to the induction of ferroptosis (7). Mitophagy, a mitochondrial quality control mechanism, selectively targets and eliminates damaged, dysfunctional, or senescent mitochondria through lysosomal degradation (8). Two major pathways drive mitophagy: the PINK1-Parkin-mediated ubiquitin pathway and the FUNDC1 receptor-mediated pathway, both of which have been extensively studied (9). Mitophagy plays a crucial role in maintaining cellular homeostasis and tumor progression. However, dysregulated mitophagy may promote tumorigenesis and tumor progression, as well as confer resistance to anticancer therapies, thereby exerting unfavorable effects (10).

In recent studies, several genes associated with mitophagy have been identified to be involved in the development of lung cancer (11). For instance, high expression of *PINK1* has been associated with poor response to chemotherapy. Studies have demonstrated a significant correlation between high *PINK1* expression and postoperative chemoresistance in lung adenocarcinoma. Therefore, *PINK1* testing could potentially aid in stratifying patients with poor chemotherapy responses and guide personalized therapy decisions (12). In addition, the depletion of *PINK1* leads to reduced ATP production, inhibition of mitophagy, and sensitization of cells to drugs targeting glycolysis in NSCLC (13). A study identified an E3 ubiquitin ligase called ARIH1/HHARI that triggers mitophagy in cancer cells in a PINK1-dependent manner. ARIH1/HHARI polyubiquitinated damages mitochondria, leading to their removal through autophagy. Moreover, *ARIH1* is widely expressed in lung adenocarcinoma and promotes chemotherapy resistance (14). Another study found that reducing *Atg7* or *Atg5*, mediated by K-RAS, in lung tumors reduced tumor burden and improved survival in malignancies associated with the rapid initiation phase of tumors (15). However, there is currently limited availability of transcriptomic and single-cell transcriptomic analyses based on mitophagy genes for the diagnosis, prognosis, and treatment of non-small cell lung cancer.

In this study, we performed a comprehensive analysis of gene expression related to mitophagy in NSCLC using the NCBI Gene Expression Omnibus (GEO). Subsequently, we developed a prediction model based on mitophagy-related signature genes to diagnose NSCLC accurately. The model was validated using an independent dataset from GEO, and NSCLC subtypes were identified through unsupervised clustering analysis. Furthermore, we performed in-depth analyses of the prognosis, immune infiltration, immunotherapy response, and drug sensitivity for each identified NSCLC subtype.

## 2 Materials and methods

### 2.1 Data acquisition and processing

We obtained RNA sequencing (RNA-seq) data and corresponding clinical information from the GEO public database (https://www.ncbi.nlm.nih.gov/geo/) for a total of 695 NSCLC samples. The obtained data underwent standardization using the R package “limma”. Among these samples, a training cohort consisting of 539 merged samples from the GSE30219 (16) and GSE32863 (17) series matrices were selected. To mitigate batch effects within the merged samples, we used the R package “SVA” (18). Additionally, we included 156 samples from the GSE19188 (19) series matrix for external validation. Single-cell data from 11 tumor samples were also included from the GSE131907 (20). Furthermore, we retrieved the mitophagy-related gene set from the Reactome database and the inflammatory factor-related gene set from the BioCarta database.

### 2.2 Differentially expressed genes analysis

Data from the GSE30219 and GSE32863 series matrices were merged and classified into normal and tumor samples based on clinical information. We extracted MRGs from the Reactome database and generated a heat map using the R package “pheatmap”. DEGs were detected between the normal and tumor samples, with a false discovery rate (FDR) threshold of less than 0.05. Subsequently, DEGs boxplots and volcano plots were constructed using the R packages “ggplot2” and “ggpubr”. In addition, a correlation heatmap of DEGs was visualized using the R package “corrplot”.

### 2.3 Analysis of scRNA-seq data in NSCLC

We performed analyses of scRNA-seq data using the R packages “Seurat” and “SingleR” (21, 22). To retain high-quality scRNA-seq data, we applied a series of filtering steps on the raw matrix. Specifically, we included only genes expressed in at least three cells, excluded cells expressing fewer than 200 genes, and removed cells with mitochondrial gene expression exceeding 8%. First, we applied the NormalizeData function to normalize the scRNA-seq data and then used the FindVariableFeatures function to identify the top 2,000 highly variable genes. Subsequently, we performed principal component analysis (PCA) using the RunPCA function from the “Seurat” package, reducing the dimensionality of the scRNA-seq data based on the top 2,000 highly variable genes. We used the ElbowPlot analysis to identify significant principal components and performed cell clustering analysis using the top 10 principal components obtained from PCA. The FindNeighbors function was employed to construct a k-nearest neighbor graph based on the Euclidean distance in the PCA space, and the UMAP algorithm was applied for data dimensionality reduction and visualization. We used reference data from the human primary cell atlas (23) for cluster annotation and the CellMarker database (CellMarker (xbio.top)) (24) for reference-based cell annotation.

To evaluate the gene expression scores of MRGs for each cell in the scRNA-seq dataset, we employed the AddModuleScore function to compute specific scores for individual cells. Furthermore, we performed GSVA to assess the enrichment of signature pathways for each cell type using the MSigDB database (http://www.gsea-msigdb.org/) (25). Following this, we performed GSEA on the differentially expressed genes between the high and low groups based on mitophagy scores (26).

### 2.4 Construction and validation of a prediction model

Machine learning models have proven to be powerful tools in handling large datasets and facilitating rapid analysis and decision-making in disease diagnosis. In our study, we employed a combined approach using support vector machine (SVM) (27) and random forest (RF) (28) algorithms to select relevant features from a set of 25 genes associated with mitophagy. Subsequently, a prediction model was constructed using logistic regression (29). The sensitivity and specificity of the prediction model were assessed through the analysis of receiver operating characteristic (ROC) curves. The performance of the model was evaluated by calculating the area under the curve (AUC) of the ROC curve. To visualize the relative importance of the feature genes within the model and their contribution to the prediction outcomes, a nomogram model was generated using the “rms” package in R (30). The prediction performance of the model was further evaluated by plotting calibration curves and decision Curve Analysis (DCA) curves. An external dataset was employed to validate the robustness of the results (31).

### 2.5 Analysis based on the human protein atlas database

To validate the prediction model, the HPA database (proteinatlas.org) was used to ascertain if the expression levels of the feature genes in NSCLC differ from those in normal tissues at the protein level.

### 2.6 Cell culture and qRT- PCR analysis

The bronchial epithelial cell BEAS‐2B and the NSCLC cell lines A549, PC9, and H1299 were from the Cell Bank of Type Culture Collection of Shanghai Institute of Biochemistry & Cell Biology, Chinese Academy of Science. BEAS‐2B cells were cultured in Gibco LHC basal medium (Invitrogen, Carlsbad, CA) supplemented with 10% fetal bovine serum, while the NSCLC cell lines were cultured in Gibco RPMI‐1640 medium (Invitrogen, Carlsbad, CA) supplemented with 10% fetal bovine serum. Total RNA was extracted using TRIZOL reagent (Invitrogen) and then reverse-transcribed into cDNA. β‐actin was used as an internal control. Real‐time PCR was performed using a Roche LightCycler® 480 System. Relative expression levels were calculated as ratios normalized against β‐actin. The QPCR primer sequences are listed in **Supplementary Table S1.**

### 2.7 Correlation between feature genes and immunity

To explore the correlation between feature genes and immunity, we employed the “CIBERSORT” package to evaluate the extent of immune cell infiltration in both normal and tumor samples. Furthermore, we performed Spearman’s correlation analysis using the “IOBR” and “psych” packages to investigate the relationships among feature genes, immune infiltration, and inflammatory factors. The results were visually represented using the “ggplot2” package (32, 33).

### 2.8 Consensus clustering analysis

We performed unsupervised clustering analysis on 351 samples from patients with NSCLC using the “ConsensusClusterPlus” R package based on the expression profiles of six feature genes. The K-means algorithm was employed with a maximum number of subtypes set to 10 (k=10), a sampling rate of 0.8, and 100 resampling iterations. The optimal number of clusters was determined by evaluating the cumulative distribution function (CDF) curve and CDF delta area curves (34).

Following the clustering analysis, we investigated the correlation between the expression levels of MRGs and clinical pathological features within different subtypes of NSCLC. In addition, using the gene set variation analysis (GSVA) package, we calculated the scores of each sample relative to the MRGs. The significance of the differential expression of MRGs between the two groups of NSCLC subtypes was assessed using the Wilcoxon test (35). Furthermore, Kaplan-Meier survival analysis was performed on the different subtypes using the “survival” and “survminer” R packages to evaluate their impact on prognosis.

### 2.9 Analysis of immune infiltration in NSCLC subtypes

In this study, we employed the CIBERSORT software code in R to evaluate the proportions of 22 immune cell subtypes within the different subtypes of NSCLC. The LM22 gene expression matrix, which includes 547 genes and defines the composition of 22 immune cell types, served as a standardized reference for characterizing the human immune cell types. By applying the CIBERSORT algorithm, we obtained the profiles of the immune cell infiltrations for each NSCLC subtype. Additionally, we used the “IOBR” package in R to estimate the expression levels of the immune and stromal cells in the samples. A comparison of the abundance of immune cells and stromal cells among different subtypes was performed, and the results were visually presented using box plots (32).

### 2.10 Weighted gene co-expression network analysis

We performed consensus module analysis using the “WGCNA” package in R to identify hub genes within each subtype (36). Initially, we ranked genes based on their median absolute deviation (MAD) values and selected the top 5000 genes. Next, we performed sample and gene filtering to exclude those with excessive missing values. A similarity matrix was constructed, which was then converted into an adjacency matrix using a weight coefficient β = 6. Subsequently, the blockwiseModules function was employed to perform modular analysis on the adjacency matrix, enabling the establishment of a gene co-expression network and the assignment of genes to different modules. For each module, module eigengenes (MEs) were computed, and their Pearson’s correlation coefficients with the phenotype data were calculated. Finally, the correlation between the module genes and phenotype data was visualized using a heat map.

### 2.11 Analysis of enrichment

Based on the WGCNA analysis results, we selected genes from the module with the highest correlation to different NSCLC subtypes. Subsequently, we performed enrichment analyses using the Metascape database (https://metascape.org/) (37). In addition, we imported gene sets from the biological processes (BP) category using the “msigdbr” package in R (38) and performed biological process enrichment analysis for different NSCLC subtypes using the “fgsea” package.

### 2.12 Prediction of immune therapy response

First, we analyzed immune checkpoint gene expression in various subtypes of NSCLC. Subsequently, we employed the Tumor Immune Dysfunction and Exclusion (TIDE) database (Tumor Immune Dysfunction and Exclusion (TIDE) (harvard.edu)) to predict the response to immune checkpoint blockade. TIDE can analyze two mechanisms involved in tumor immune evasion: one causing dysfunction of cytotoxic T lymphocyte (CTL) infiltration and the other impeding CTL infiltration into the tumor, known as “exclusion” (39, 40). The use of TIDE facilitated a more effective prediction of the therapeutic efficacy of immunotherapy.

### 2.13 Drug sensitivity analysis

The half-maximal inhibitory concentration (IC50) is a commonly used concentration indicator to assess drug toxicity or therapeutic efficacy. We used the “oncoPredict” package in R and employed expression matrices from the Genomics of Drug Sensitivity in Cancer (GDSC) database, along with drug treatment information, as a training set to evaluate chemotherapy sensitivity in different subtypes of NSCLC (41).

### 2.14 Statistical analysis

All the statistical analyses were performed by R-4.1.3. Student's t-test or Wilcoxon’s rank sum test was used to detect the significant difference between two independent groups, p < 0.05 was considered statistically significant.

## 3 Results

### 3.1 Upregulation of MRGs and activation of the immune system in NSCLC

**Figure 1:**
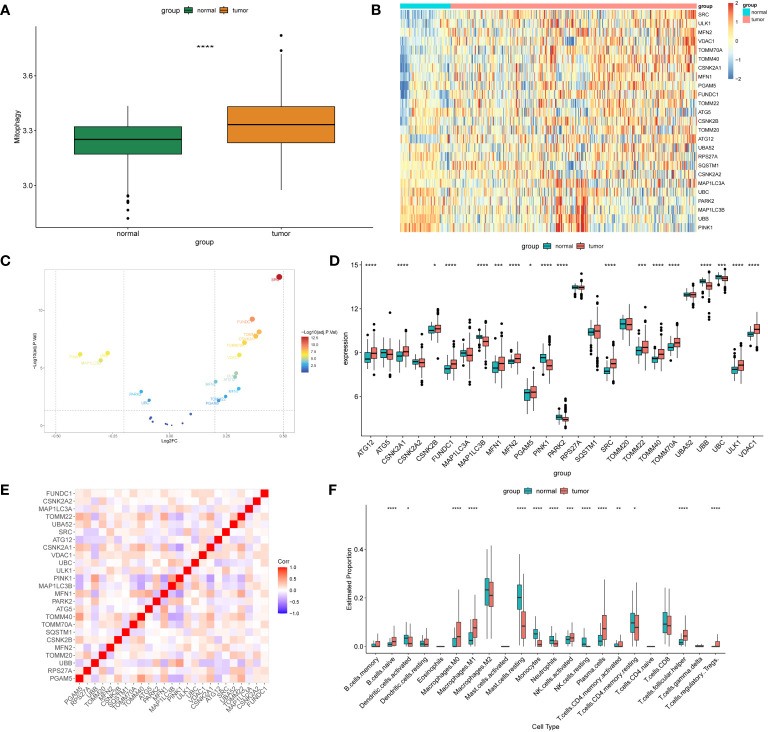
Differentially expressed MRGs in NSCLC. **(A)** The box plot displays the GSVA scores of the MRG gene set across samples to observe the overall expression differences of MRG between healthy individuals and patients with NSCLC. P-values were estimated by Wilcoxon rank-sum test. ****P < 0.0001. **(B)** The expression patterns of 25 MRGs are presented in the heatmap. Each row represents a specific MRG and each column represents a sample. There are a total of 351 tumor samples and 72 normal samples. The color gradient ranging from blue to red indicates low to high expression levels, respectively. **(C)** The volcano map illustrates 25 differentially expressed MRGs in NSCLC, where a negative log2FC indicates downregulation and a positive log2FC indicates upregulation of the gene expression. **(D)** The box plot displays the differential expression of 25 MRGs between healthy individuals and NSCLC patients. P-values were estimated by Wilcoxon rank-sum test. *P < 0.05, *** P < 0.001, ****P < 0.0001. **(E)** Correlation analysis between the 25 differentially expressed genes. Red and blue represent positive and negative correlations, respectively. The depth of color represents different correlation coefficients. **(F)** The box plot shows the expression differences of 22 immune cells between healthy individuals and NSCLC patients. P-values were estimated by Wilcoxon rank-sum test. *P < 0.05, ** P < 0.01, *** P < 0.001, ****P < 0.0001.

We performed batch correction and merged the GSE30219 and GSE32863 datasets to investigate the expression profiles of MRGs in the combined dataset. Our analysis revealed that the expression levels of MRGs were significantly lower in normal tissues than in tumor tissues (**Figure 1A**). Among the 25 MRGs exhibiting differential expression between the normal and tumor tissues, only *MAP1LC3B*, *PINK1*, *PARK2*, *UBB*, and *UBC* were significantly downregulated in the tumor tissues, while expression of the other genes was significantly upregulated in the tumor samples (**Figures 1B–D**). Furthermore, correlation analysis demonstrated the associations among the 25 differentially expressed genes related to mitophagy (**Figure 1E**). Using CIBERSORT analysis, the results showed a significant increase in the infiltration of naive B cells, M0 and M1 macrophages, NK cells, plasma cells, helper T cells, and regulatory T cells in the tumor tissues (**Figure 1F**).

### 3.2 Expression of MRGs based on scRNA-seq data

**Figure 2:**
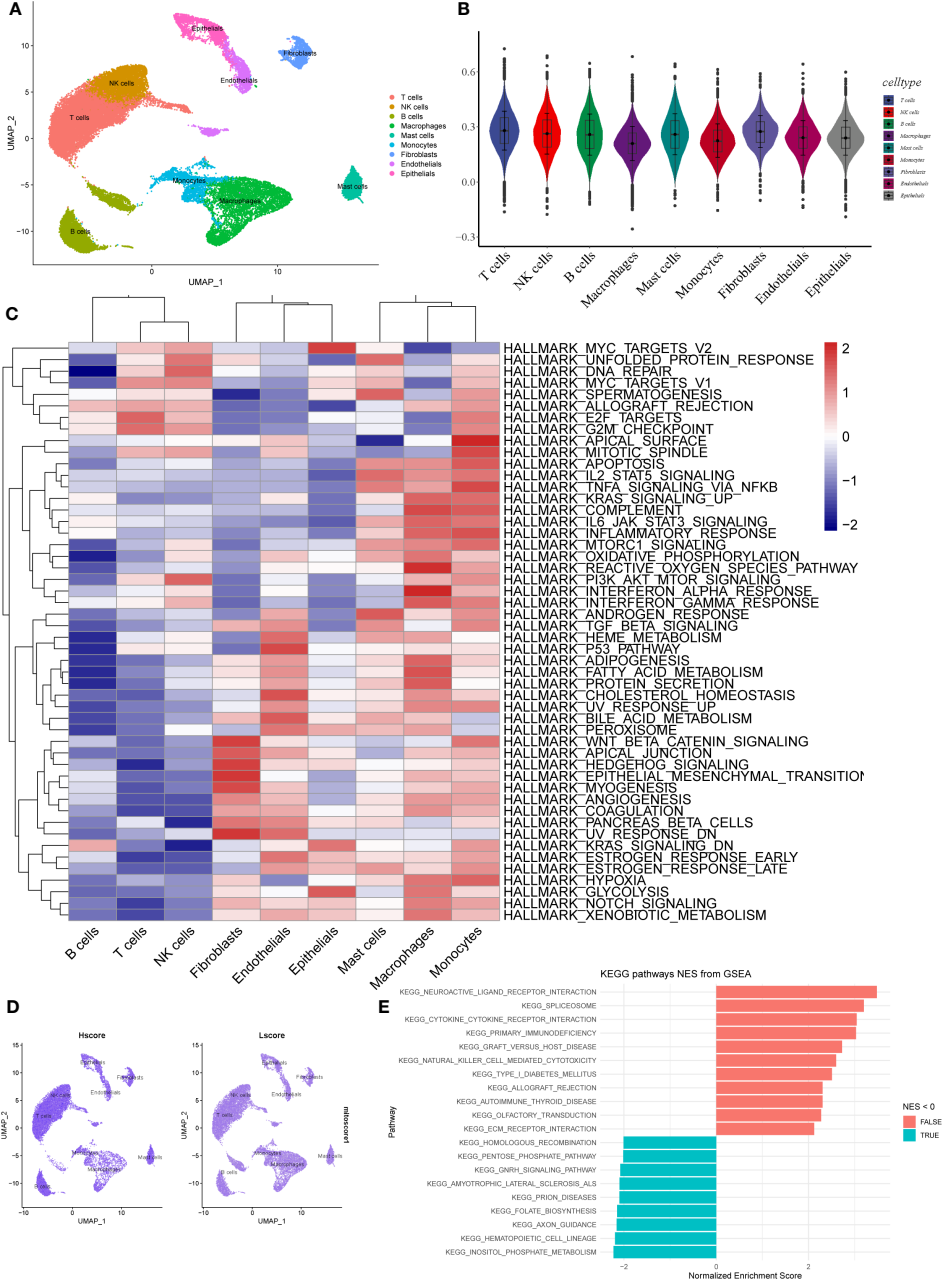
Expression of MRGs based on scRNA-seq data. **(A)** The cells were clustered into 9 types using the UMAP dimensionality reduction algorithm, and each color represents the annotation phenotype of each cluster. **(B)** Scoring MRGS in various cell types based on AddModuleScore. **(C)** The heatmap displays the enrichment of different pathways identified through GSVA analysis in different cell types. The color gradient ranging from blue to red indicates low to high expression levels, respectively. **(D)** The left panel shows the scoring of MRGS in various cell types for the high-scoring group, while the right panel shows the low-scoring group. **(E)** The bar plot displays the pathways enriched with differentially expressed genes between the high and low-scoring groups of mitochondrial autophagy. The red color indicates NES > 0, indicating pathways associated with high expression, while the blue color represents pathways associated with low expression.

Based on the scRNA-seq data from GSE131907, we performed quality control on 11 tumor samples in the single-cell dataset (**Supplementary Figure 1A**) to ensure the quality of the cells included in the study. Then, the top 2,000 highly variable genes were identified for PCA to reduce dimensionality (**Supplementary Figures 1B, C**). Subsequently, we clustered the cells into 21 clusters (**Supplementary Figure 1D**). We classified them roughly into T cells, NK cells, macrophages, monocytes, mast cells, epithelial cells, endothelial cells, B cells, and fibroblasts based on the results of singleR and CellMarkers database (**Supplementary Figures 1E, F**, **Figure 2A**). The AddModuleScore function was used to calculate the score of the different cell types based on the MRGs. We found that fibroblasts had the highest MRG score (mitoscore), followed by mast cells (**Figures 2B–D**). We then performed GSVA analysis on different cells and found that the HALLMARK inflammation-related pathways such as apoptosis, inflammatory_response, TGF_BETA_SIGNALING, and TNFA_SIGNALING_VIA_NFKB were upregulated in mast cells (**Figure 2C**). In addition, a GSEA analysis was performed between samples with an Hscore and Lscore for mitophagy-related scores. We found that the differentially expressed genes between the high and low groups were highly enriched in pathways such as KEGG_neuroactive_ligand_receptor_interaction, KEGG_cytokine_cytokine_receptor_interaction, KEGG_natural_killer_cell_mediated_cytotoxicity, and KEGG_ecm_receptor_interaction (**Figure 2E**).

### 3.3 Construction and validation of a prediction model

**Figure 3:**
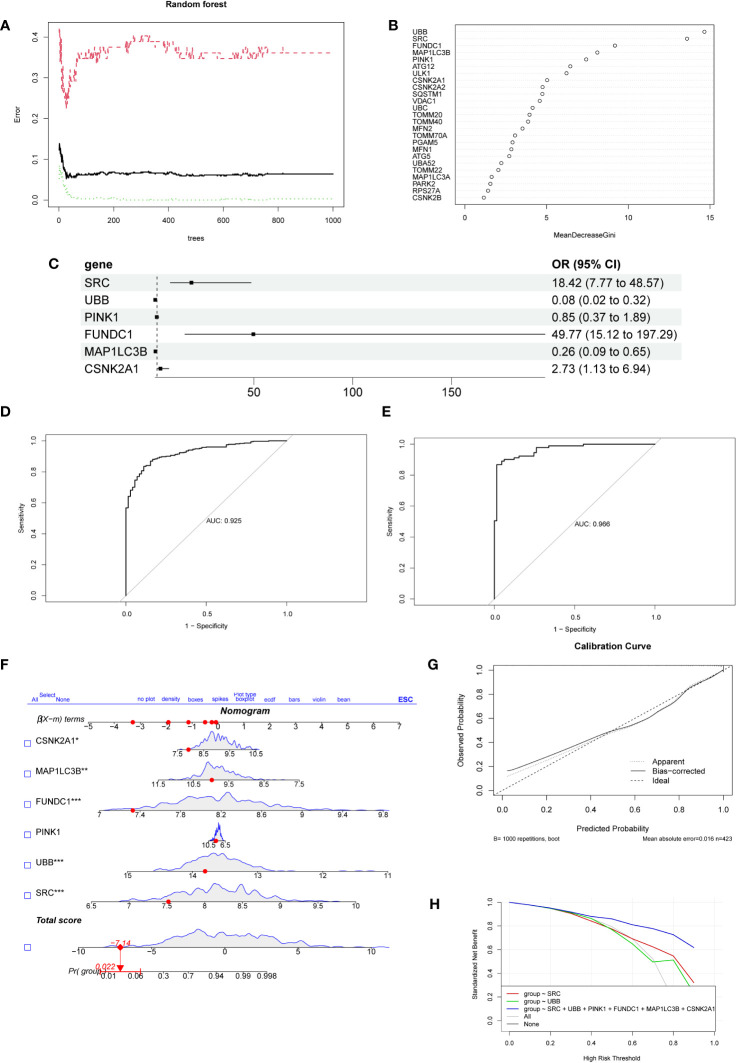
The establishment and evaluation of the diagnostic model. **(A)** The error rate curve of the Random Forest model. The plot demonstrates the variation of the prediction error of the Random Forest learning model under different quantities of trees. **(B)** The ranking plot displays the importance scores of genes as evaluated by the Random Forest model. The MeanDecreaseGini index is used to measure the importance of feature genes in the model, where a higher value indicates a higher level of importance of that variable in the model.**(C)** The forest plot displays the 6 genes selected through logistic regression.**(D)** Receiver operating characteristic curve evaluating the diagnostic performance of feature genes. The horizontal axis represents the false positive rate, while the vertical axis represents the true positive rate. **(E)** The receiver operating characteristic curve was used to evaluate the diagnostic performance of the feature genes in the external validation dataset GSE19188. **(F)** Nomogram for predicting the risk of NSCLC based on feature genes. The feature weight of six important feature genes is used as input variables, and the “Total points” denote the total score obtained by adding up the scores of all input variables. “Pr(group)” represents the risk score corresponding to the “Total points,” which indicates the likelihood of developing NSCLC. **(G)** Calibration curve illustrating the calibration performance of a predictive model. The horizontal axis represents the predicted value, while the vertical axis represents the actual observed value. The closer the bias-corrected curve is to the Ideal dashed line, the higher the calibration performance of the model. **(H)** DCA estimates the clinical benefit of the nomogram. The plot shows a comparison of the net clinical benefits for different prediction models at different decision thresholds. The model constructed using six feature genes exhibits the highest clinical benefit.

We used SVM and RF algorithms to select feature genes from the 25 MRGs and predict the occurrence of NSCLC. The SVM analysis allowed us to identify the top 10 optimal feature genes associated with NSCLC occurrence. Subsequently, we performed a random forest analysis, which involved selecting the top 10 genes based on the error rate curve. The gene importance scores were obtained from the random forest model (**Figures 3A, B**). The genes selected by the SVM-RFE algorithm and random forest were intersected to obtain the final set of genes (*SRC*, *UBB*, *PINK1*, *FUNDC1*, *MAP1LC3B*, *CSNK2A1*). Multivariable logistic regression analysis demonstrated that these six selected genes were associated with NSCLC (**Figure 3C**). We further evaluated the predictive performance of the model using an ROC curve, and the area under the curve (AUC) was determined to be 0.925. In addition, we validated the model using an external dataset (GSE19188), and the AUC based on this validation was 0.966 (**Figures 3D, E**). To visually illustrate the prediction performance of the feature genes, we created a nomogram model (**Figure 3F**). The model, which integrated the information from all six feature genes, showed superior prediction value compared to individual feature genes. To assess the calibration and clinical utility of the model, we generated calibration plots and DCA curve analysis (**Figures 3G, H**). These analyses demonstrated that the nomogram model had good prediction efficacy for NSCLC. Subsequently, we collected IHC staining images of six feature genes-associated proteins from the HPA database, which were obtained from both NSCLC and normal lung tissue. Significantly higher protein expression levels were observed for three of the feature genes (*SRC*, *CSNK2A1, FUNDC1*) in NSCLC samples relative to normal samples, thereby supporting our findings (**Supplementary Figure 2A**). The protein expression levels of the additional three feature genes showed no significant difference between the NSCLC samples and the normal samples. Furthermore, qPCR was used to validate the expression of six feature genes that were screened in both the lung cancer cell lines (A549, PC9, H1299) and the normal bronchial epithelial cell line (BEAS-2B). Results indicated a significant upregulation of the six feature genes in lung cancer cells (**Supplementary Figure 2B**). The expression patterns of *CSNK2A1*, *FUNDC1*, and *SRC* were consistent with our bioinformatics analysis results, therefore, we attempted to construct a prediction model using these three genes. The predictive performance of the model based on the three feature genes was not as good as that of the six-gene model, as evidenced by the ROC curve (**Supplementary Figure 2C**), and the AUC based on this model was only 0.882. In addition, we analyzed the correlation between the six feature genes and immune cells, as well as inflammatory factors. Among the selected feature genes, *UBB*, *PINK1*, and *MAP1LC3B* exhibited a negative correlation with the infiltration of the aforementioned immune cells, while the other feature genes showed a positive correlation (**Supplementary Figure 3A**). *UBB*, *PINK1*, and *MAP1LC3B* were significantly positively correlated with 10 out of 24 inflammatory factors, whereas the remaining feature genes showed the opposite trend (**Supplementary Figure 3B**).

### 3.4 Identification and differential analysis of subtypes in NSCLC

**Figure 4:**
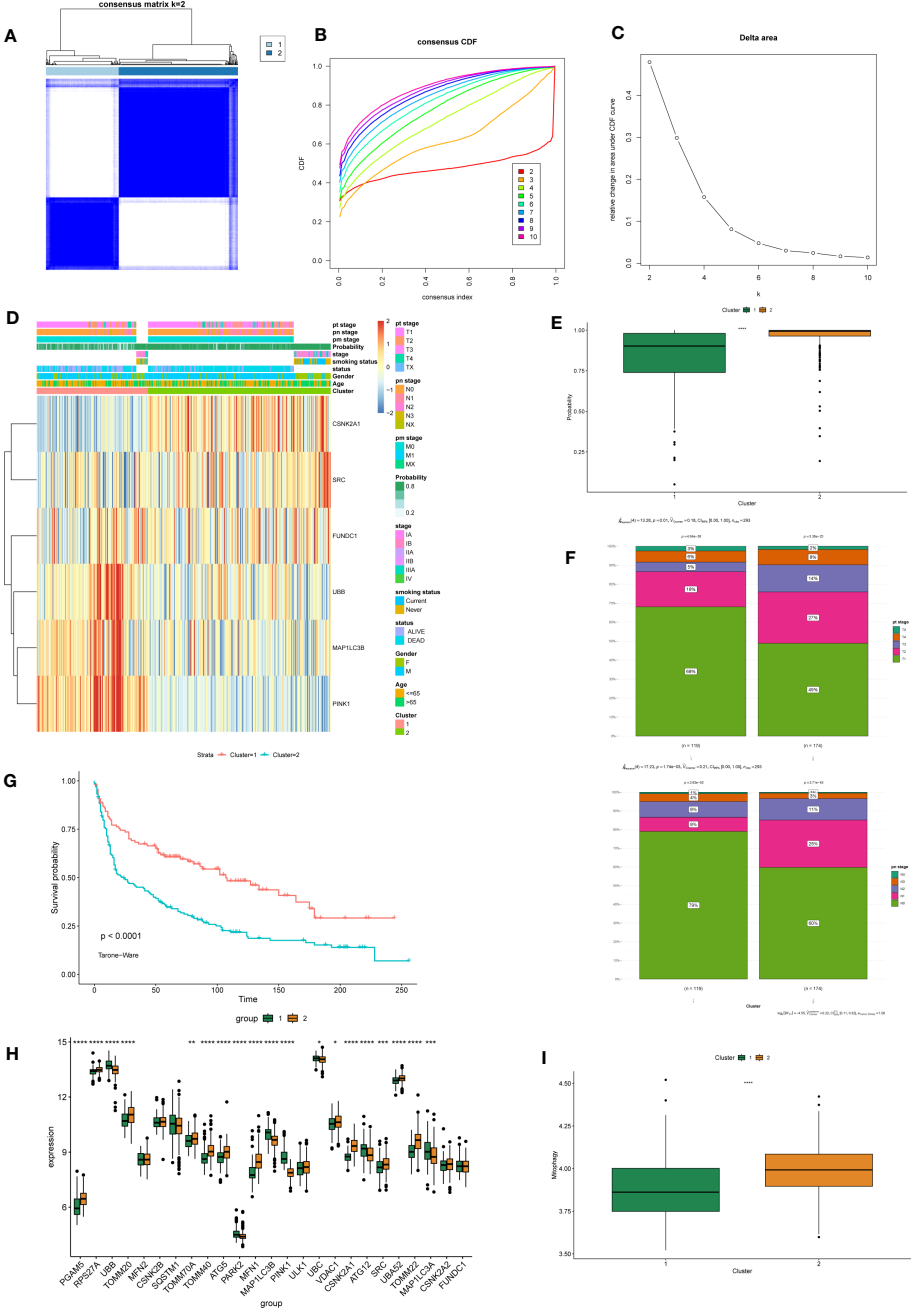
Identification and Differential Analysis of mitophagy-related NSCLC subtypes. **(A)** Consensus clustering matrix showing the clustering agreement between samples when k (number of clusters) = 2. **(B)** Representative cumulative distribution function (CDF) curve showing the clustering results for k (number of clusters) ranging from 2 to 10. **(C)** Relative changes in CDF delta area curves, which measure the stability of clustering across different values of k (number of clusters). **(D)** The heatmap displays the expression of mitochondrial autophagy-related feature genes with clinical information in different subtypes of NSCLC. **(E)** The box plot compares the diagnostic performance of prediction models for two subtypes of NSCLC. P-values were estimated by Wilcoxon rank-sum test. ****P < 0.0001. **(F)** The stacked bar chart illustrates the distribution differences of tumor T and N staging between two subtypes of NSCLC. P-values were estimated by Student's t-test. **(G)** The survival curves demonstrate the survival differences between the two subtypes of NSCLC. The horizontal axis represents the survival time, and the vertical axis represents the survival probability. The p-values were calculated using the Tarone-Ware test. **(H)** The box plot illustrates the differential expression of 25 MRGs between two subtypes of NSCLC. P-values were estimated by Wilcoxon rank-sum test. *P < 0.05, ** P < 0.01, *** P < 0.001, ****P < 0.0001. **(I)** The box plot displays the GSVA scores of the MRG gene set in two subtypes of NSCLC. P-values were estimated by Wilcoxon rank-sum test. ****P < 0.0001.

Based on the six feature genes related to mitochondrial autophagy, unsupervised clustering was performed to classify all tumor samples into subtypes. Through comprehensive analysis of the cumulative distribution function (CDF) curve (**Figure 4B**) and delta area (**Figure 4C**), the optimal segmentation efficiency was achieved at k=2, with a clustering number of 2 (**Figure 4A**). The heat map illustrates the differences in expression levels of the feature genes and the distribution of clinical pathological features between the two subtypes (**Figure 4D**). We observed that the prediction model demonstrated good efficacy for both subtypes of NSCLC (**Figure 4E**). Cluster 1 had a significantly higher number of individuals in tumor stages T1–T2 and N0–N1 than Cluster 2 (**Figure 4F**). Survival analysis also demonstrated a significantly higher survival rate in Cluster 1 than in Cluster 2 (**Figure 4G**). GSVA analysis revealed a significantly higher overall expression level of MRGs in Cluster 2 than in Cluster 1 (**Figures 4H, I**).

### 3.5 Constructing a gene co-expression network

**Figure 5:**
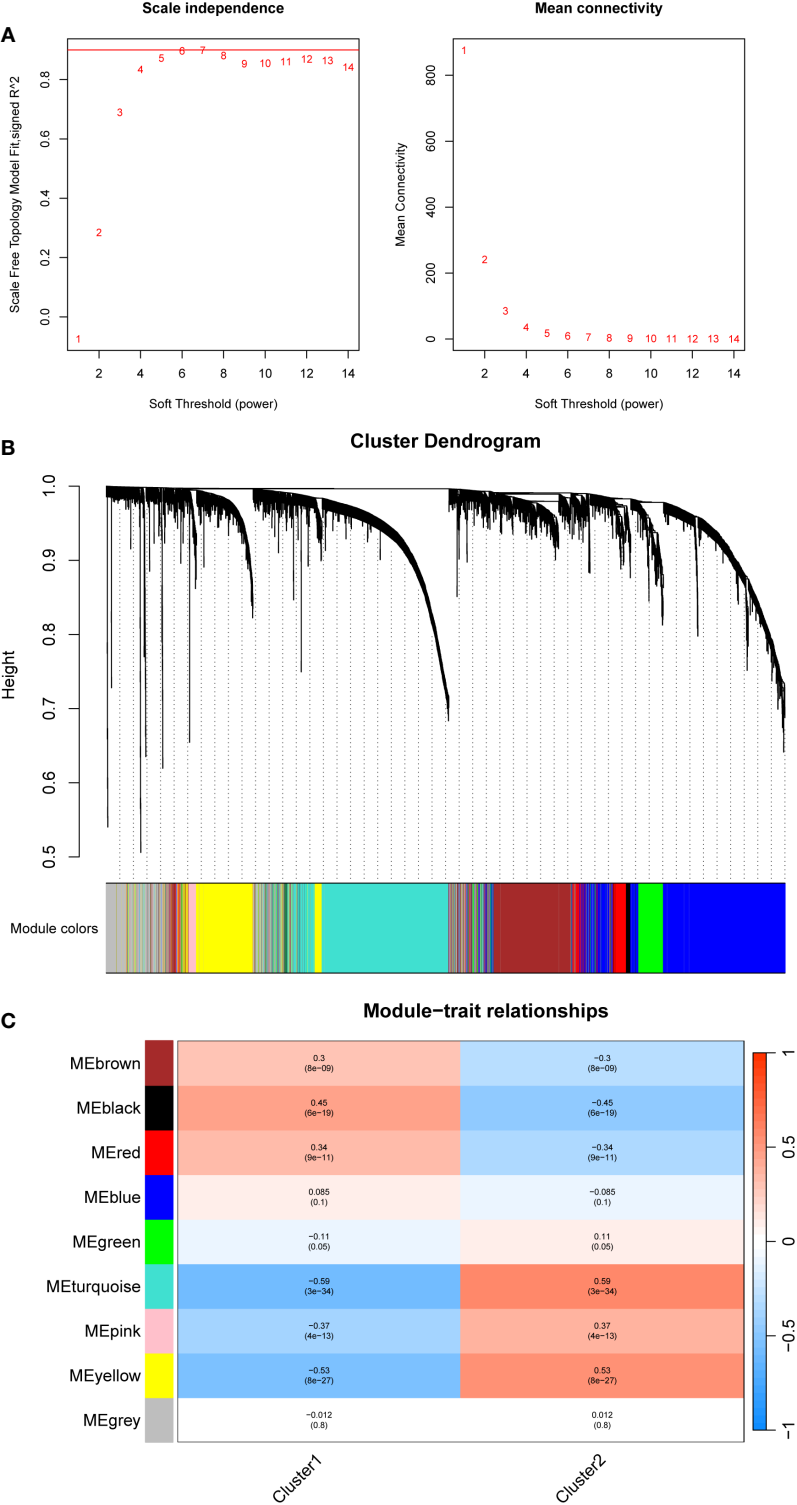
Constructing a gene co-expression network using WGCNA in different mitophagy-related NSCLC subtypes patients. **(A)** The scale plot of WGCNA to identify optimal vector power (cutoff value = 0.85). **(B)** Sample dendrogram and trait heatmap. **(C)** Correlations between different modules and clusters: every module has its correlation coefficient and corresponding p-value.

**Figure 6:**
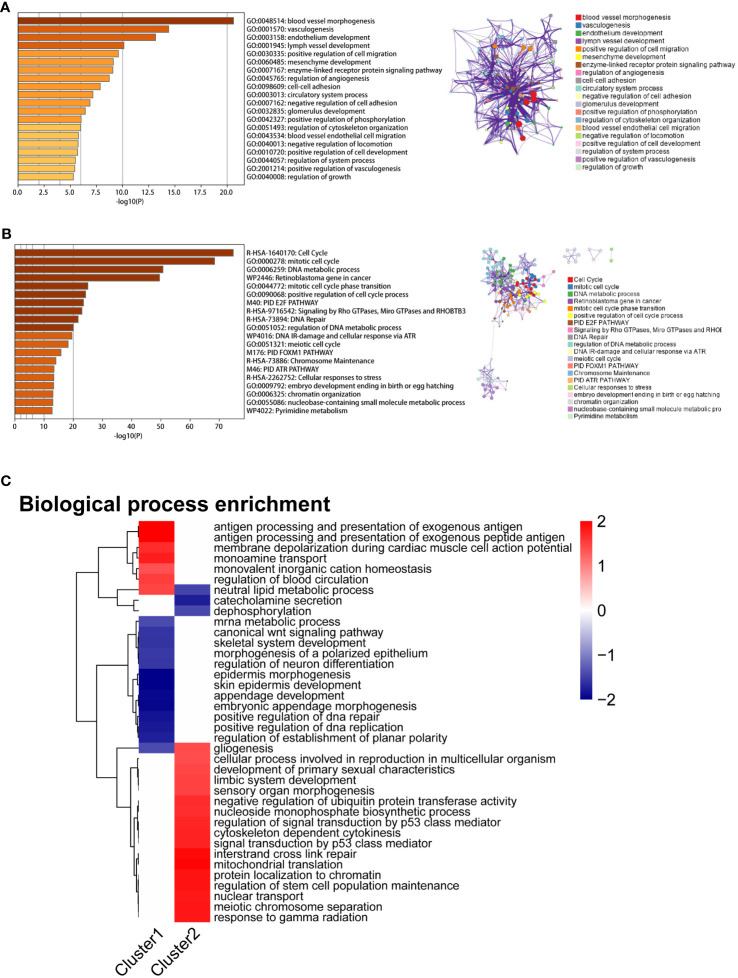
Enrichment analysis results of two mitophagy-related NSCLC subtypes. **(A)** The bar graph displays the enrichment results of hub genes associated with the MEblack module, which exhibits the highest correlation with Cluster 1. Colored by p-values. **(B)** The bar graph displays the enrichment results of hub genes associated with the MEturquoise module, which exhibits the highest correlation with Cluster 2. Colored by p-values. **(C)** The heatmap illustrates the Fgsea results of Cluster1 and Cluster2. The color gradient ranging from blue to red indicates low to high expression levels, respectively.

A weighted gene co-expression network analysis (WGCNA) was performed to identify co-expression modules that showed the highest correlation with each cluster. WGCNA was performed on both datasets of diseased samples after batch normalization. All samples were included in the analysis, and the optimal soft-thresholding value was set to 6. The results revealed a total of nine modules generated (**Figures 5A, B**). Among these modules, the black module and the turquoise module exhibited the strongest correlation with Cluster 1 and Cluster 2, respectively (**Figure 5C**). Subsequently, we performed functional enrichment analysis on the WGCNA modules that exhibited the highest correlation with each cluster. For this analysis, we used the Metascape database to analyze the genes within the black module and the turquoise module. The results revealed significant enrichment in the pathways associated with angiogenesis, endothelial development, and cell adhesion in Cluster 1 (**Figure 6A**). In Cluster 2, there was significant enrichment in the pathways related to cell cycle and metabolism (**Figure 6B**). Furthermore, FGSEA highlighted that Cluster 1 was enriched in the pathway of antigen processing and presentation of exogenous antigens. On the other hand, Cluster 2 exhibited enrichment in pathways such as signal transduction by p53 class mediator and mitochondrial translation (**Figure 6C**).

### 3.6 Predicting the immune therapy response and sensitivity to chemotherapy drugs in different mitophagy-related NSCLC subtypes

**Figure 7:**
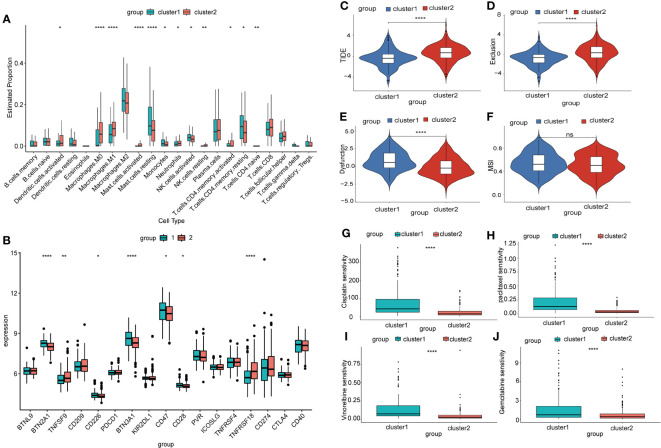
Prediction of immune therapy outcomes and sensitivity to chemotherapy drugs in different mitophagy-related NSCLC subtypes. **(A)** The box plot illustrates the immune infiltration profiles of two subtypes of NSCLC assessed using CIBERSORT. P-values were estimated by Wilcoxon rank-sum test. *P < 0.05, ** P < 0.01, ****P < 0.0001. **(B)** The box plot displays the differential expression of immune checkpoint genes in two subtypes of NSCLC. P-values were estimated by Wilcoxon rank-sum test. *P < 0.05, ** P < 0.01, ****P < 0.0001. **(C)** The box plot depicts the prediction of the TIDE score in two subtypes of NSCLC based on the TIDE database, which is a metric used to assess tumor immune function and predict the response to immunotherapy. P-values were estimated by Wilcoxon rank-sum test. ***P < 0.001. **(D)** Exclusion score is a metric used to assess the degree of immune exclusion phenomenon within tumors. P-values were estimated by Wilcoxon rank-sum test. ***P < 0.001. **(E)** Dysfunction score is a metric used to measure the degree of abnormality in tumor immune function. P-values were estimated by Wilcoxon rank-sum test. ***P < 0.001. **(F)** MSI score is a measure used to assess the genomic stability of a tumor and detect the level of microsatellite instability within it. P-values were estimated by Wilcoxon rank-sum test. ***P < 0.001. **(G)** The sensitivity of two mitophagy-related NSCLC subtypes to cisplatin is shown in box plots with IC50 values on the y-axis. P-values were estimated by Wilcoxon rank-sum test. ***P < 0.0001. **(H)** The sensitivity of two mitophagy-related NSCLC subtypes to paclitaxel is shown in box plots with IC50 values on the y-axis. P-values were estimated by Wilcoxon rank-sum test. ***P < 0.0001. **(I)** The sensitivity of two mitophagy-related NSCLC subtypes to vincristine is shown in box plots with IC50 values on the y-axis. P-values were estimated by Wilcoxon rank-sum test. ***P < 0.0001. **(J)** The sensitivity of two mitophagy-related NSCLC subtypes to gemcitabine is shown in box plots with IC50 values on the y-axis. P-values were estimated by Wilcoxon rank-sum test. ***P < 0.0001.

To investigate the differences in the immune microenvironment among different subtypes of NSCLC, we used the CIBERSORT package and IOBR package to assess the infiltration proportions of distinct immune and stromal cells. The infiltration levels of dendritic cells, M1 macrophages, mast cells, neutrophils, fibroblasts, and T cell CD4 memory cells were significantly elevated in Cluster 2, while monocytes, NK cells, and endothelial cells exhibited higher infiltration levels in Cluster 1 (**Figure 7A**, **Supplementary Figure 4A**). The expression of most inflammatory factors was higher in Cluster 1 than in Cluster 2 (**Supplementary Figure 4B**).

Immunotherapy checkpoint inhibitors have demonstrated durable efficacy in some NSCLC patients. In this study, we investigated the differences in expression levels of immunotherapy checkpoint genes between two NSCLC subtypes. Our analysis revealed significant differences in the expression levels of several immunotherapy checkpoint genes, including *BTN2A1*, *TNFSF9*, *CD226*, *BTN3A1*, *CD47*, *CD28*, and *TNFRSF18* (**Figure 7B**). This suggested that these two NSCLC subtypes might respond differently to immunotherapy checkpoint inhibitors. We further employed the TIDE algorithm to predict the effectiveness of immunotherapy checkpoint inhibition treatment. We observed that Cluster 1 exhibited a significantly lower TIDE score (**Figure 7C**), indicating a better response to immunotherapy. In addition, we found that Cluster 1 had a lower Exclusion score and a higher Dysfunction score compared to other clusters. However, there was no significant difference in the microsatellite instability (MSI) score between the clusters (**Figures 7D–F**). To explore the sensitivity to chemotherapy drugs in different NSCLC subtypes, we used the oncoPredict package to evaluate the IC50 values of several chemotherapy drugs. The results of the drug sensitivities showed that the IC50 values of cisplatin, paclitaxel, vincristine, and gemcitabine were lower in Cluster 2 than in Cluster 1. This meant that patients in Cluster 2 might benefit more from the above chemotherapy drugs (**Figures 7G–J**). Additionally, our correlation analysis between drug response and gene expression revealed that the highly expressed MRGs in Cluster 2 negatively correlate with the IC50 values of the aforementioned chemotherapy drugs (**Supplementary Figures 5A-D**).

## 4 Discussion

Studies have consistently demonstrated the involvement of mitophagy in various cancer processes, including tumor initiation, progression (42), augmentation of immunotherapy (43), and enhancement of chemotherapy sensitivity (44). However, most investigations have focused primarily on individual MRGs, and research using comprehensive mitophagy-related gene sets remains limited. In our study, GSVA analysis revealed a significant upregulation in the overall expression of mitophagy-related gene sets within the tumor samples. Therefore, we developed a prediction model for NSCLC based on six carefully selected mitophagy-related feature genes. The model exhibited remarkable efficacy in assisting NSCLC diagnosis, as evidenced by an impressive area under the curve (AUC) value of 0.925 for diagnosis efficiency within the GEO queue and an AUC of 0.966 during external validation. Furthermore, leveraging these six feature genes, we successfully stratified NSCLC patients into two distinct subtypes, which displayed significant differences in prognosis. A comprehensive analysis confirmed that these subtypes also exhibited notable disparities in immune infiltration, chemotherapy response, and immunotherapy potential. Extending our investigation to single-cell analysis of NSCLC provided additional insights into the intricate relationship between mitophagy, inflammation, and immunity in NSCLC. These findings have shed new light on the underlying mechanisms driving NSCLC, ultimately contributing to a broader understanding of this complex disease.

The prediction model consisted of six mitophagy-related genes (*SRC*, *UBB*, *PINK1*, *FUNDC1*, *MAP1LC3B*, and *CSNK2A1*). It was constructed through machine learning using SVM and random forest algorithms for feature selection. Analysis revealed a strong correlation between these six genes and the infiltration of certain immune cells, indicating their potential role in immune regulation within tumors. *SRC* is a tyrosine kinase involved in multiple aspects of tumor development, including proliferation, migration, and angiogenesis. It was highly expressed in the NSCLC samples. The inhibition of *SRC* has emerged as a feasible therapeutic strategy for treating advanced NSCLC (45). In addition, studies have revealed elevated expression levels of *FUNDC1* in lung cancer tissues, which aligns with our findings (46). Research has indicated that high expression of *PINK1* is associated with poor chemotherapy response. Elevated *PINK1* expression is significantly correlated with postoperative chemoresistance in lung adenocarcinoma (12). Silencing *PINK1* can inhibit the proliferation of lung cancer cells and disrupt their cell cycle (47). Furthermore, *PINK1* and *Parkin* have been proposed as tumor suppressor factors. *MAP1LC3B*, also known as *LC3B*, is a protein involved in autophagy and mitophagy. It participates in the formation and fusion of autophagosomes, facilitating the degradation and clearance of cellular waste materials. Interestingly, research has suggested that high expression of *LC3B* is associated with lower invasiveness of NSCLC tumors (48). *CSNK2A1* is a gene that encodes the protein kinase CK2α, which regulates various cellular functions, including cell proliferation, apoptosis, DNA repair, cell cycle control, and transcriptional regulation. Research has shown that knocking down *CSNK2A1* in KRAS (G12C) mutant lung cancer cells reduced cell proliferation, inhibited Wnt/β-catenin signaling, and enhanced the anti-proliferative effects of MEK inhibitors on KRAS (G12C) mutant lung cancer cells (49). *UBB* is a gene that encodes ubiquitin B, a protein involved in the ubiquitin-proteasome system. Ubiquitin is overexpressed in NSCLC, and targeting *UBB* and *UBC* genes in NSCLC H1299 cells to inhibit ubiquitin expression leads to suppressed cell growth. Furthermore, inhibiting ubiquitin expression has been observed to increase cellular radiosensitivity (50). Therefore, these mitophagy-related genes played a crucial role in the development and progression of NSCLC, and they may have clinical significance as potential biomarkers.

Our research classified NSCLC patients into two subtypes based on the expression of MRGs, and there were significant differences in MRGs expression between the two subtypes. The overall expression of MRGs in Cluster 2 was significantly higher than in Cluster 1. Among the six featured genes, *PINK1*, *MAP1LC3B*, and *UBB* exhibited significantly higher expression in Cluster 1, while *SRC* and *CSNK2A1* showed significantly higher expression in Cluster 2. Studies have shown a significant correlation between elevated *PINK1* expression and postoperative chemoresistance in lung adenocarcinoma (51). This is consistent with our analysis of chemotherapy sensitivity in the two subtypes. Cluster 1, characterized by high *PINK1* expression, exhibited poorer chemotherapy sensitivity than Cluster 2. Furthermore, Cluster 2 had a higher proportion of patients in tumor stages T2–T3 and M1–M2, and survival analysis indicated that Cluster 1 had significantly better overall survival compared to Cluster 2. Additionally, our prediction model, constructed based on MRGs expression, demonstrated significantly higher prediction performance for Cluster 2 with overall higher expression compared to Cluster 1.

The tumor microenvironment, particularly the immune microenvironment, plays a crucial role in the recurrence and metastasis processes of NSCLC. It imposes significant limitations on the efficacy of immunotherapy and chemotherapy (52, 53). The infiltration of immune cells plays a significant role in the progression, metastasis, and immune escape of NSCLC (54). Our research identified significant differences in immune cell infiltration between healthy individuals and patients with NSCLC. Specifically, the infiltration proportions of mast cells, B cells, and M1 macrophages were significantly increased in NSCLC patients. Furthermore, the different subtypes of NSCLC exhibited variations in the proportions of immune cell infiltration. Cluster 2 had a notably higher proportion of infiltrated mast cells, dendritic cells, and neutrophils. In contrast, Cluster 1 showed higher infiltration proportions of monocytes, NK cells, and resting CD4 T cell memory. Mast cells serve as biomarkers and critical determinants of cancer treatment response, and in lung cancer, mast cell density is associated with angiogenesis and poor prognosis (55, 56). In addition, studies have found that neutrophils promoted cancer angiogenesis by releasing vascular endothelial growth factor (VEGF) and other pro-angiogenic factors. Neutrophils are the primary source of VEGFA expression in the tumor microenvironment of NSCLC (57). These findings are consistent with our results, as patients in Cluster 2 with high overall expression of mitophagy exhibited a poorer prognosis. Based on the evaluation of immune cell infiltration using MCPcounter, we found that Cluster 1 exhibited a significantly higher abundance of T cells than Cluster 2. In addition, immune checkpoint analysis revealed higher expression levels of immune checkpoints on T cells in Cluster 1 compared to Cluster 2. These results suggested that patients in Cluster 1 would benefit more from immunotherapy than patients in Cluster 2. Furthermore, our analysis based on the TIDE algorithm, which predicts the efficacy of immunotherapy, also showed that Cluster 1 patients were more suitable for immunotherapy than Cluster 2 patients.

Chemoresistance poses a major obstacle in the treatment of NSCLC. Distinguishing individuals who would be sensitive to chemotherapy could maximize the effectiveness of NSCLC treatment. In our study, we performed chemosensitivity analysis on patients with different subtypes of NSCLC using the Genomics of Drug Sensitivity in Cancer (GDSC) database. We found that patients in Cluster 2 with high expression levels of mitophagy exhibited increased sensitivity to drugs such as paclitaxel, cisplatin, and gemcitabine. The 2023 NSCLC-NCCN guidelines also recommend the use of nivolumab plus platinum-based doublet chemotherapy as a neoadjuvant systemic treatment regimen for patients with resectable (tumor size ≥ 4 cm or positive lymph nodes) NSCLC (58). Furthermore, research has indicated a correlation between high *PINK1* expression and poor response to chemotherapy in NSCLC (51). These findings align with our results of elevated *PINK1* expression in Cluster 1, while Cluster 2 appeared to be more responsive to chemotherapy. Our drug sensitivity analysis provided new insight into the relationship between mitochondrial autophagy and systemic treatment strategies for NSCLC.

WGCNA was used to identify hub genes associated with each subtype by screening for modules with the highest correlation. The identified hub genes were then subjected to enrichment analysis using the Metascape database. In Cluster 1, enrichment analysis revealed pathways related to angiogenesis, cell migration, cell adhesion, and endothelial development, indicating that patients in this cluster may be more prone to tumor progression and migration. We found that Cluster 2, characterized by high expression of mitophagy, was associated with pathways related to cell cycle and metabolism. Previous research has indicated that during mitophagy, the activation of *TBK1* mediated by *PINK1* and *Parkin* led to mitotic arrest, thereby influencing the cell cycle. On the other hand, the loss of *PINK1* and *PRKN* allows the cell cycle to proceed (59), meeting the metabolic demands of the tumor and promoting the progression of NSCLC. These findings are consistent with our analysis, which showed poorer survival outcomes and lower expression of *PINK1* in Cluster 2.

Based on scRNA-seq analysis, we revealed a significant correlation between MRGs and the immune microenvironment. Autophagy has a dual role in cancer. Before tumor initiation, it inhibits tumor development. However, once the tumor has started to progress, autophagy promotes tumor growth in unfavorable microenvironments (60, 61). Disruption of the immune system in NSCLC can lead to immune evasion, immune suppression, enhanced inflammatory response, and resistance to immunotherapy. We observed high expression of MRGs in both mast and T cells. In addition, the inflammatory-related pathways such as TNF_A_SIGNALING_VIA_NFKB, INFLAMMATORY_RESPONSE, and APOPTOSIS were significantly enriched in mast cells. Research has indicated that mast cells can release a substantial amount of TNF-α (62), which can lead to direct cytotoxicity against tumor cells. However, in other contexts, TNF-α promotes tumor growth (63) and creates a tumor-supportive microenvironment, ultimately facilitating tumor growth and progression. In addition, differentially expressed genes between the high-scoring and low-scoring groups of MRGs were enriched in pathways related to cell signaling, cell-cell interactions, and immune regulation. The enrichment of these pathways may result in various effects within tumors, including enhanced immune regulation and inflammatory responses, increased proliferation and survival signaling in tumor cells, and enhanced metastatic and invasive capabilities of tumor cells. These effects have significant implications for tumor initiation and progression and might provide targets for therapeutic intervention. However, the specific impacts and mechanisms of these effects require further research to enhance our comprehension.

The current study has certain limitations. First, this research is retrospective rather than prospective, leading to incomplete data information. Additionally, while we validated the feature genes and confirmed our research findings, only three genes' expressions (*CSNK2A1*, *FUNDC1*, and *SRC*) in tumor cells supported our results. The prediction model based on these three genes did not perform as well as the one based on six genes(*SRC*, *UBB*, *PINK1*, *FUNDC1*, *MAP1LC3B*, and *CSNK2A1*). To bolster the validity of our findings, an extension of the cell lines used is essential. Furthermore, more *in vitro* studies are warranted for comprehensive validation.

## 5 Conclusion

Our study used machine learning to identify six mitophagy-related feature genes, which may serve as prediction biomarkers for NSCLC patients. Furthermore, based on these six feature genes, unsupervised clustering classified NSCLC patients into two subtypes. These subtypes exhibited significant differences in prognosis, immune infiltration, and response to immunotherapy and chemotherapy. Additionally, we conducted single-cell analyses to explore the interaction between mitophagy and immunity in NSCLC. This research provided novel insights into the relationship between mitophagy and NSCLC and contributed to future investigations in this field.

## Data availability statement

The datasets [GSE30219, GSE32863, GSE19188, GSE131907] for this study can be found in the [Gene Expression Omnibus] https://www.ncbi.nlm.nih.gov/geo/. 

## Author contributions

HY: Data curation, Investigation, Methodology, Resources, Visualization, Writing – original draft. MJ: Supervision, Writing – review & editing. GH: Funding acquisition, Resources, Writing – review & editing. QC: Methodology, Supervision, Writing – review & editing. QL: Validation, Investigation, Writing – original draft.
