# Supplementary material for: Comprehensive analysis of mitophagy-related genes in NSCLC diagnosis and immune scenery: based on bulk and single-cell RNA sequencing data
Source: Front Immunol. 2023 Dec 14;14:1276074. doi: 10.3389/fimmu.2023.1276074 (PMC10752969; doi:10.3389/fimmu.2023.1276074)
Supplement: Supplementary file 1 [file DataSheet_1.pdf]

Supplementary Material

1 Supplementary Figures and Tables

1.1 Supplementary Figures

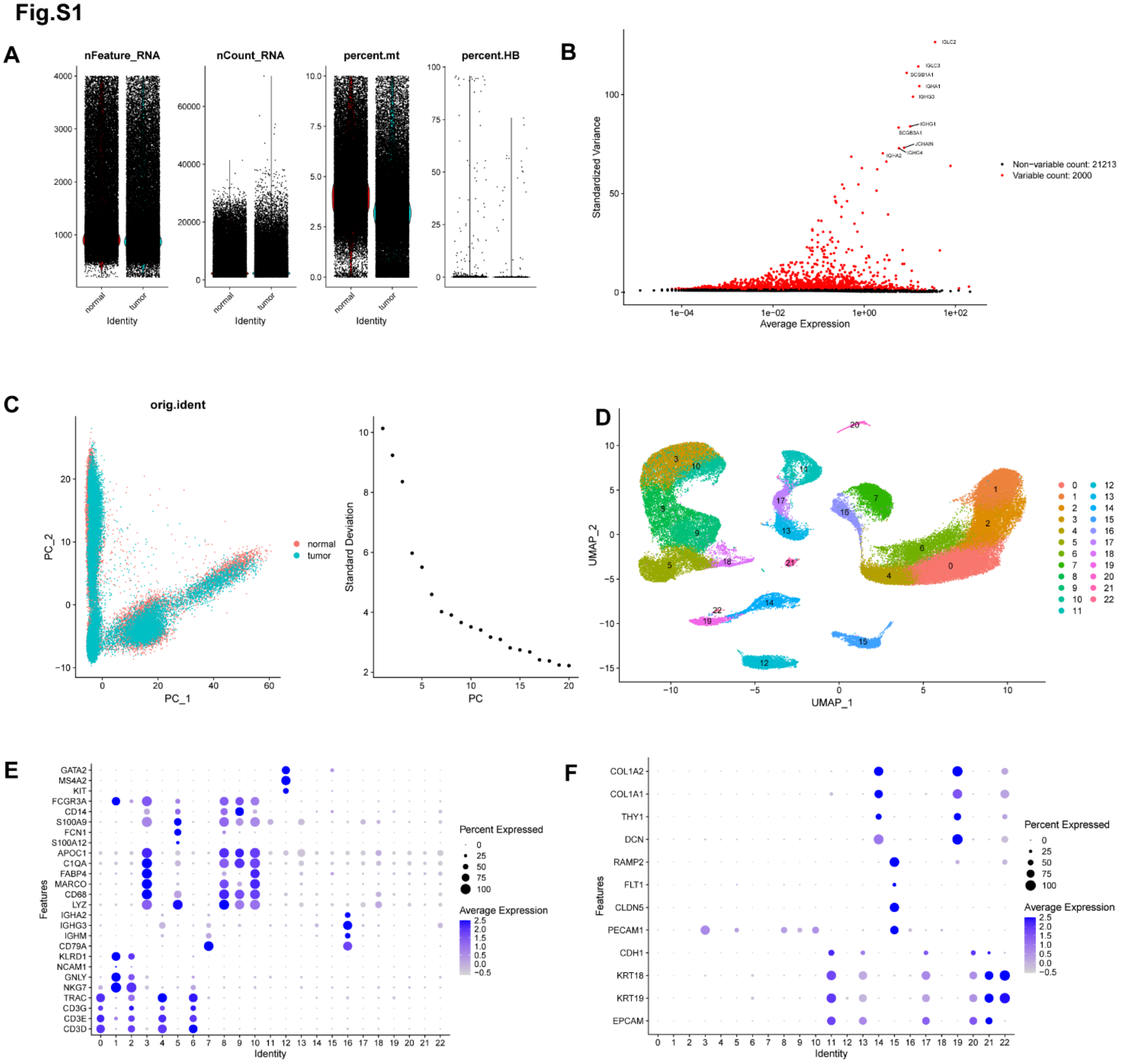

Supplementary Figure 1. Analysis of scRNA-seq data in NSCLC. (A)The number of genes per cell (nFeature_RNA), the number of unique molecular identifiers (UMI) per cell (nCount_RNA), the

percentage of mitochondrial genes per cell (percent.mt), and the percentage of hemoglobin genes per cell (percent.HB) in scRNA-seq data. (B)The variance plot displays a total of 22,568 genes in all cells, with red dots representing the top 2,000 highly variable genes. (C)After performing PCA dimensionality reduction, the elbow plot was used to select the top 10 principal components. (D)The cells were clustered into 21 clusters using UMAP for dimensionality reduction and clustering. (E)The bubble Chart displays the expression of marker genes across the 21 detected cell clusters. (F)The bubble Chart displays the expression of marker genes across the 21 detected cell clusters.

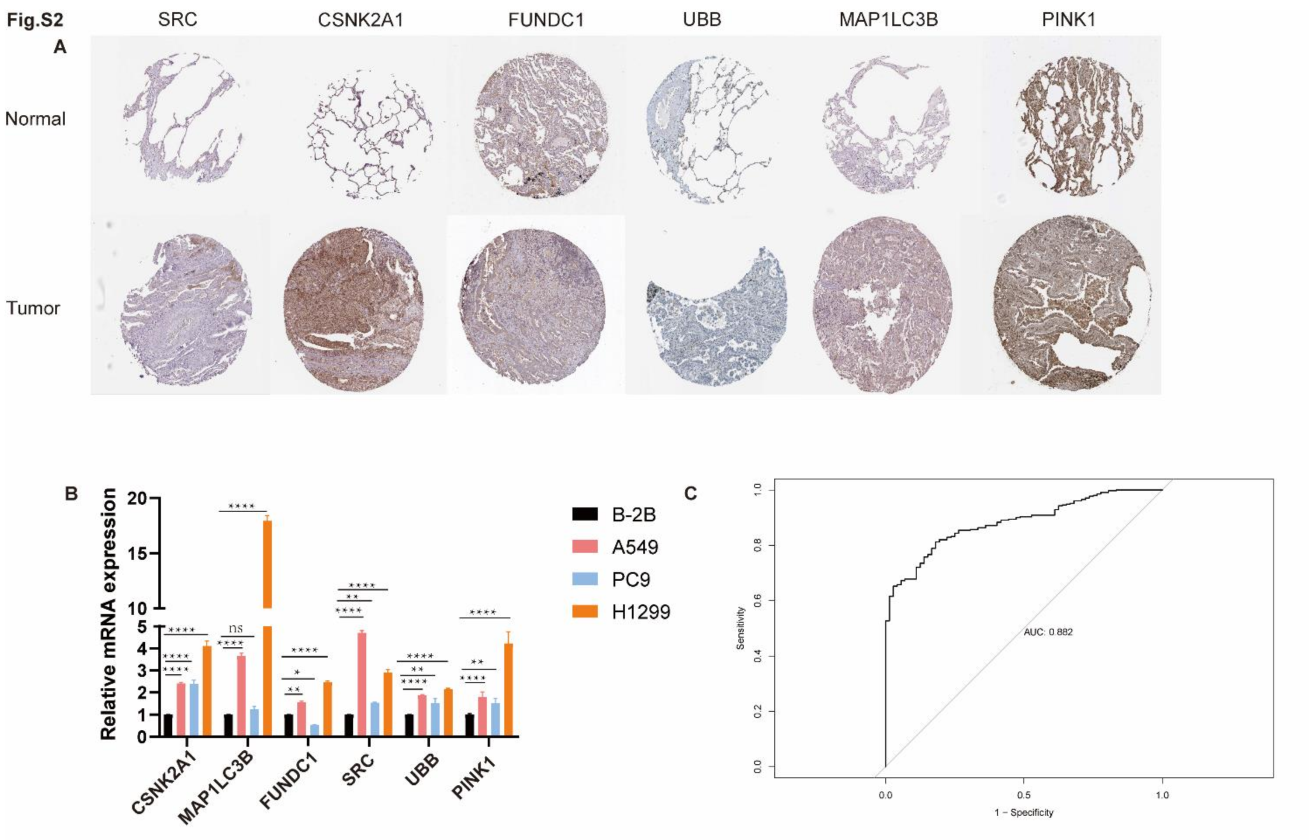

Supplementary Figure 2. Validation of the predictive model. (A)The immunohistochemical staining reveals the expression of the six feature genes at the protein level in both normal and NSCLC tissues. (B)The expression differences of six feature genes in lung cancer cell lines (A549, PC9, H1299) and the normal bronchial epithelial cell line (BEAS-2B) were assessed using qRT-PCR. P-values were estimated by Wilcoxon rank-sum test. *P < 0.05, ** P < 0.01, ****P < 0.0001, ns stands for not

significant. (C)Receiver operating characteristic curve evaluating the diagnostic performance of three feature genes (*CSNK2A1*, *FUNDC1*, and *SRC*).

Fig.S3

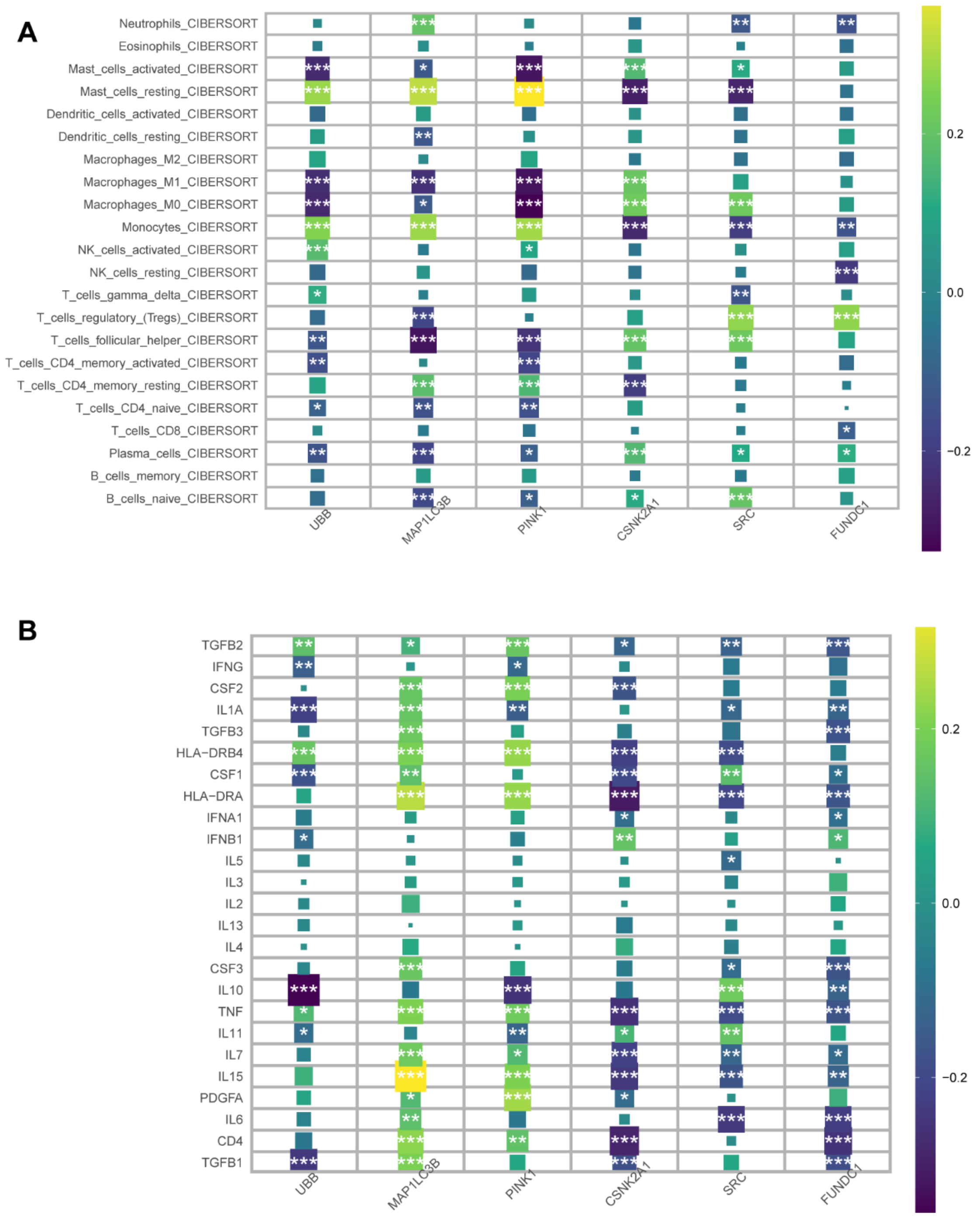

Supplementary Figure 3. Correlation between Expression of 6 Genetic Features and Immunity. (A)The heatmap depicts the correlation between the expression of feature genes and the infiltration of 22 immune cells. P-values were estimated by Wilcoxon rank-sum test. *P < 0.05, ** P < 0.01, *** P < 0.001. (B)The heatmap displays the correlation between the expression of feature genes and 25

inflammatory factors. P-values were estimated by Wilcoxon rank-sum test. *P < 0.05, ** P < 0.01, *** P < 0.001.

Fig.S4

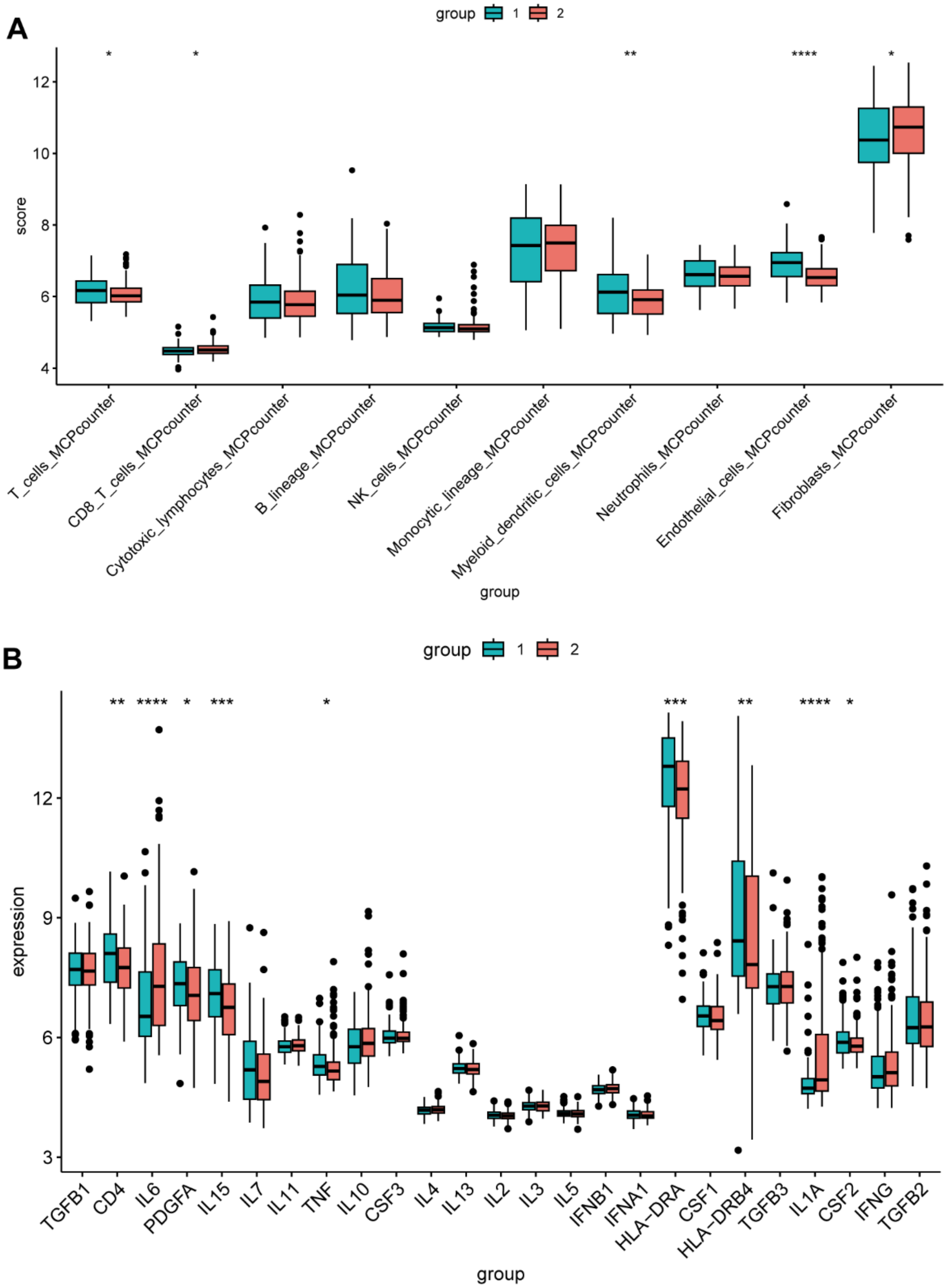

Supplementary Figure 4. Immune infiltration in mitophagy-related NSCLC subtypes. (A)The box plot displays the immune cell and stromal cell infiltration profiles of two subtypes of NSCLC evaluated using MCPcounter. P-values were estimated by Wilcoxon rank-sum test. *P < 0.05, ** P < 0.01, ****P < 0.0001. (B)The box plot depicts the differential expression of inflammatory cytokines

between two subtypes of NSCLC. P-values were estimated by Wilcoxon rank-sum test. *P < 0.05, ** P < 0.01, *** P < 0.001, ****P < 0.0001.

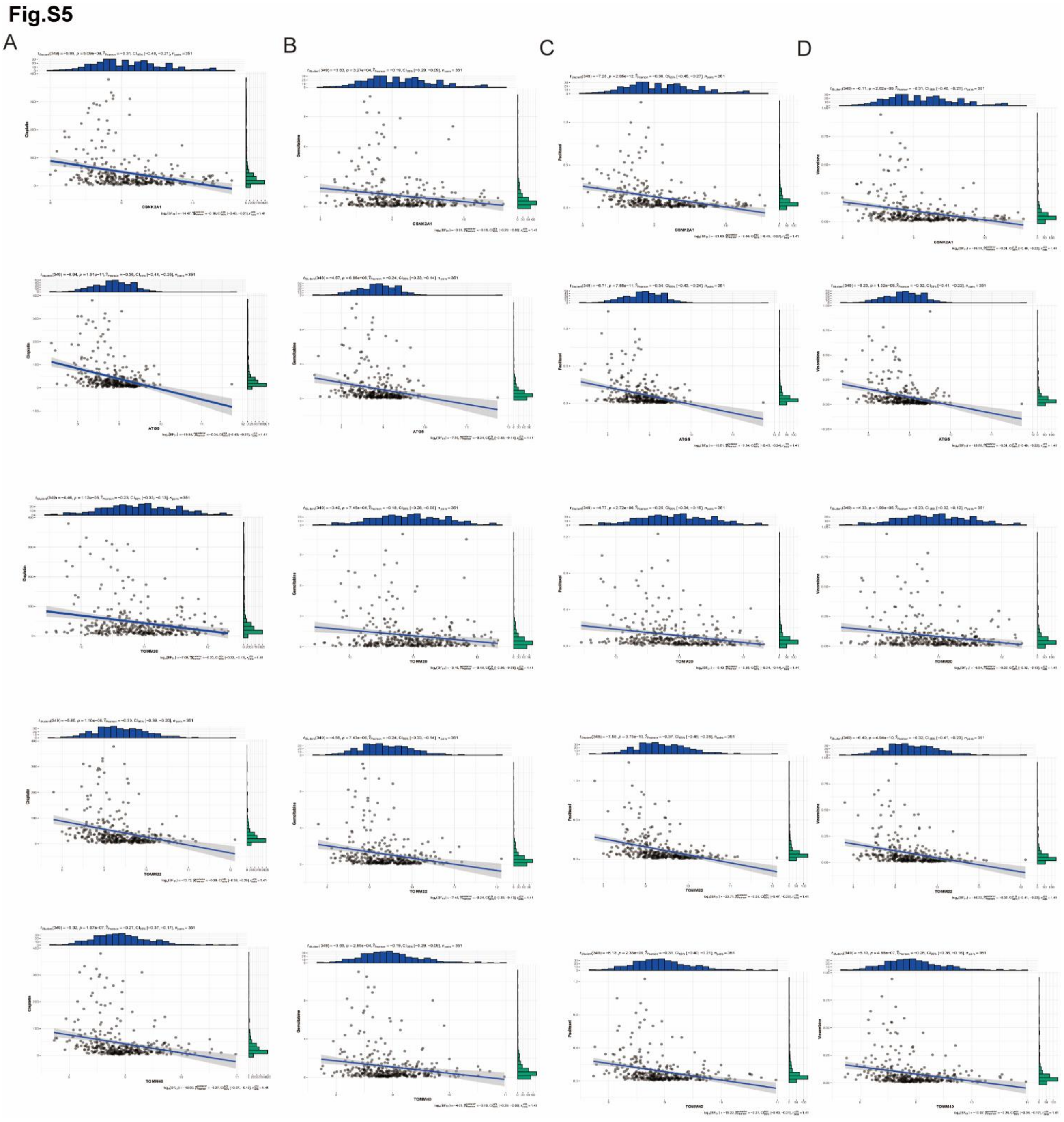

Supplementary Figure 5. Analysis of the correlation between drug sensitivity and upregulated MRGs in Cluster 2 (A, B, C, D representing cisplatin, gemcitabine , paclitaxel, and, vincristine, respectively). P-values were estimated by Wilcoxon rank-sum test.

1.2 Supplementary Tables

Supplementary Table 1. Primer sequences.

CSNK2A1	F	GAACGCTTTGTCCACAGTGA
	R	TATCGCAGCAGTTTGTCCAG
FUNDC1	F	ATGGGTGGCGTTACTGGC
	R	TGCTTTGTTCGCTCGTTT
SRC	F	CTCTATGGCCGCTTCACCAT
	R	GGTTCACCATCCCAGGGTAG
MAP1LC3B	F	AACGGGCTGTGTGAGAAAAC
	R	AGTGAGGACTTTGGGTGTGG
PINK1	F	GAGCAGACTCCCAGTTCTCG
	R	GTCCCACTCCACAAGGATGT
UBB	F	CTTTGTTGGGTGAGCTTGTTTGT
	R	GACCTGTTAGCGGATACCAGGAT
